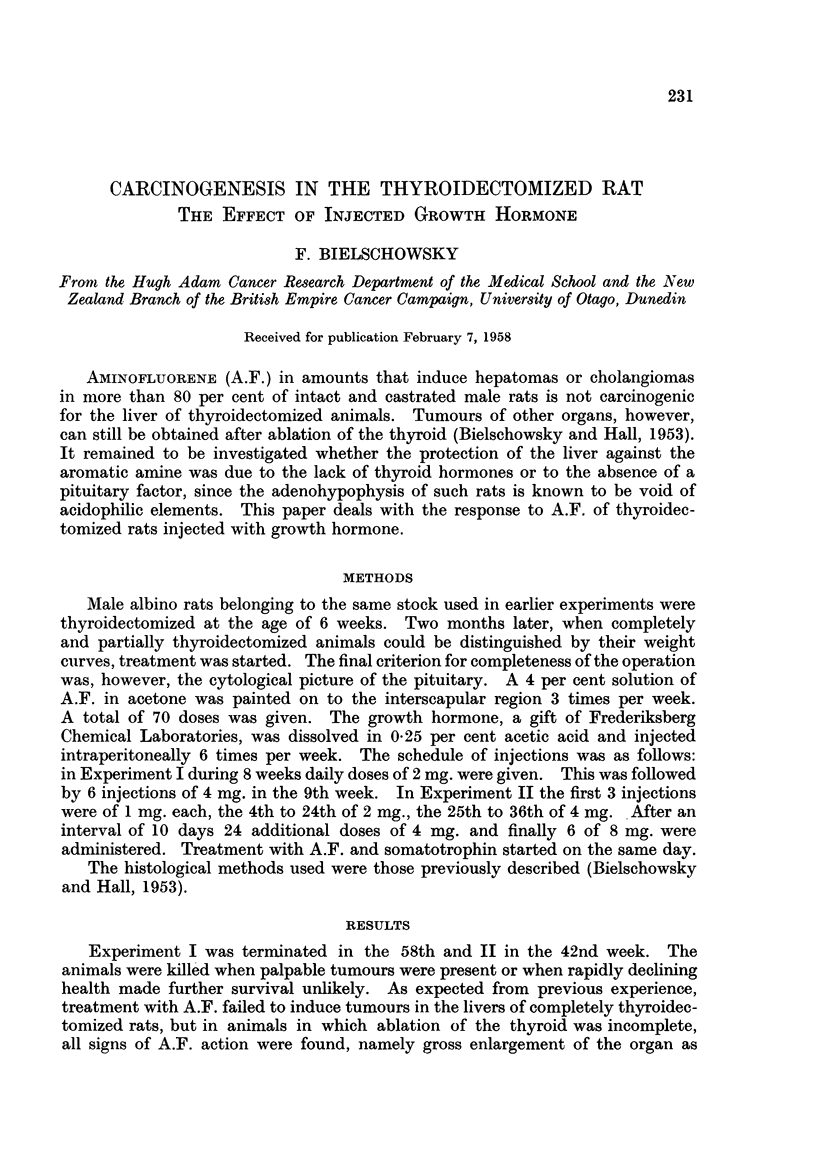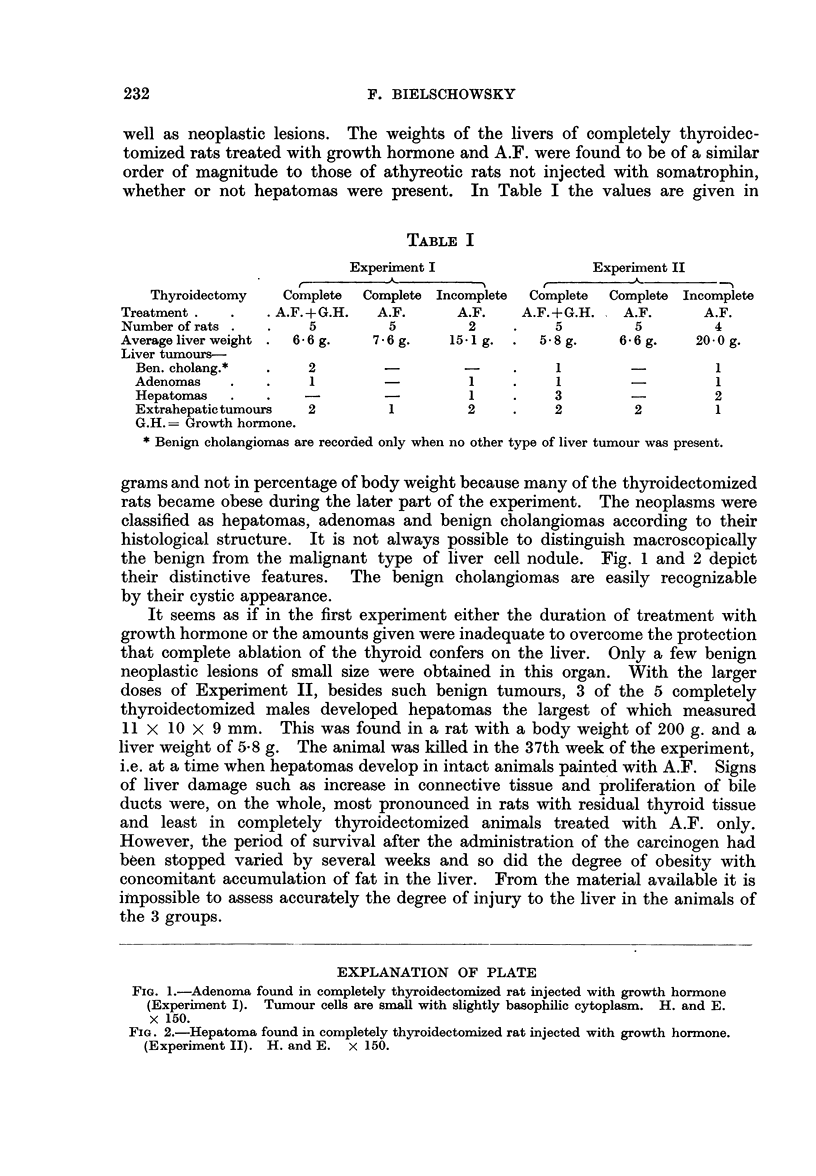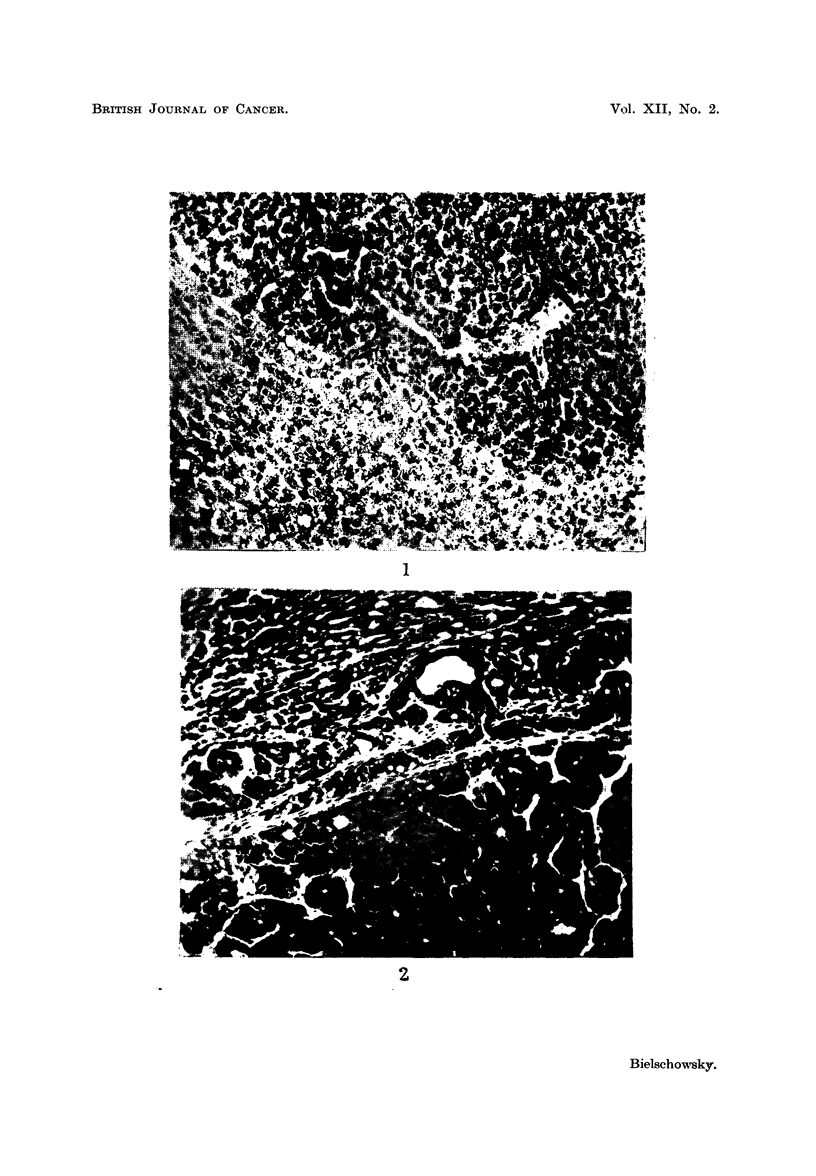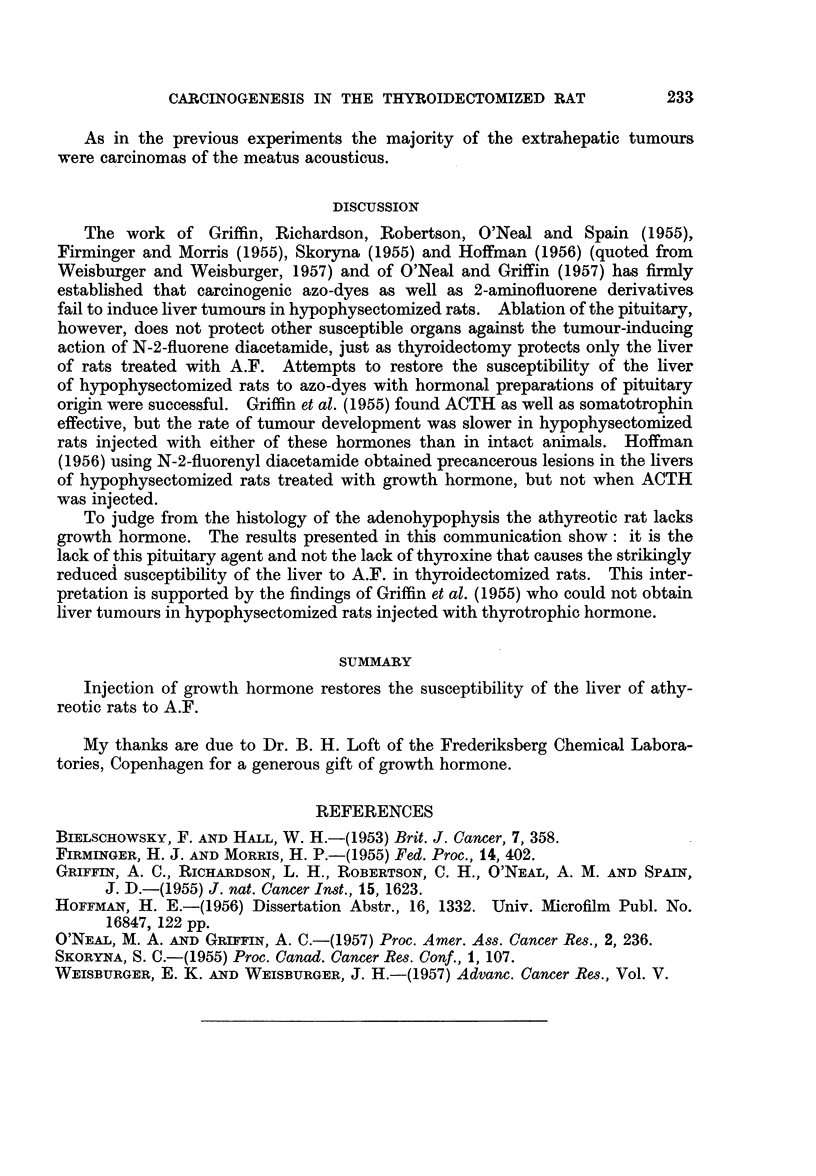# Carcinogenesis in the Thyroidectomized Rat

**DOI:** 10.1038/bjc.1958.27

**Published:** 1958-06

**Authors:** F. Bielschowsky

## Abstract

**Images:**


### CARCINOGENESIS IN THE THYROIDECTOMIZED RAT

### THE EFFECT OF INJECTED GROWTH HORMONE

F. BIELSCHOWSKY

*From the Hugh Adam Cancer Research Department of the Medical School and the New Zealand Branch of the British Empire Cancer Campaign, University of Otago, Dunedin*

Received for publication February 7, 1958

AMINOFLUORENE (A.F.) in amounts that induce hepatomas or cholangiomas in more than 80 per cent of intact and castrated male rats is not carcinogenic for the liver of thyroidectomized animals. Tumours of other organs, however, can still be obtained after ablation of the thyroid (Bielschowsky and Hall, 1953). It remained to be investigated whether the protection of the liver against the aromatic amine was due to the lack of thyroid hormones or to the absence of a pituitary factor, since the adenohypophysis of such rats is known to be void of acidophilic elements. This paper deals with the response to A.F. of thyroidectomized rats injected with growth hormone.

### METHODS

Male albino rats belonging to the same stock used in earlier experiments were thyroidectomized at the age of 6 weeks. Two months later, when completely and partially thyroidectomized animals could be distinguished by their weight curves, treatment was started. The final criterion for completeness of the operation was, however, the cytological picture of the pituitary. A 4 per cent solution of A.F. in acetone was painted on to the interscapular region 3 times per week. A total of 70 doses was given. The growth hormone, a gift of Frederiksberg Chemical Laboratories, was dissolved in 0·25 per cent acetic acid and injected intraperitoneally 6 times per week. The schedule of injections was as follows: in Experiment I during 8 weeks daily doses of 2 mg. were given. This was followed by 6 injections of 4 mg. in the 9th week. In Experiment II the first 3 injections were of 1 mg. each, the 4th to 24th of 2 mg., the 25th to 36th of 4 mg. After an interval of 10 days 24 additional doses of 4 mg. and finally 6 of 8 mg. were administered. Treatment with A.F. and somatotrophin started on the same day.

The histological methods used were those previously described (Bielschowsky and Hall, 1953).

### RESULTS

Experiment I was terminated in the 58th and II in the 42nd week. The animals were killed when palpable tumours were present or when rapidly declining health made further survival unlikely. As expected from previous experience, treatment with A.F. failed to induce tumours in the livers of completely thyroidectomized rats, but in animals in which ablation of the thyroid was incomplete, all signs of A.F. action were found, namely gross enlargement of the organ as well as neoplastic lesions. The weights of the livers of completely thyroidectomized rats treated with growth hormone and A.F. were found to be of a similar order of magnitude to those of athyreotic rats not injected with somatrophin, whether or not hepatomas were present. In Table I the values are given in

TABLE I

| | Experiment I | | | Experiment II | | |
|---|---|---|---|---|---|---|
| Thyroidectomy | Complete | Complete | Incomplete | Complete | Complete | Incomplete |
| Treatment . . . | A.F.+G.H. | A.F. | A.F. | A.F.+G.H. | A.F. | A.F. |
| Number of rats . . | 5 | 5 | 2 | 5 | 5 | 4 |
| Average liver weight . | 6·6 g. | 7·6 g. | 15·1 g. | 5·8 g. | 6·6 g. | 20·0 g. |
| Liver tumours— | | | | | | |
| Ben. cholang.* . | 2 | — | — | 1 | — | 1 |
| Adenomas . . | 1 | — | 1 | 1 | — | 1 |
| Hepatomas . . | — | — | 1 | 3 | — | 2 |
| Extrahepatic tumours | 2 | 1 | 2 | 2 | 2 | 1 |

G.H. = Growth hormone.

* Benign cholangiomas are recorded only when no other type of liver tumour was present.

grams and not in percentage of body weight because many of the thyroidectomized rats became obese during the later part of the experiment. The neoplasms were classified as hepatomas, adenomas and benign cholangiomas according to their histological structure. It is not always possible to distinguish macroscopically the benign from the malignant type of liver cell nodule. Fig. 1 and 2 depict their distinctive features. The benign cholangiomas are easily recognizable by their cystic appearance.

It seems as if in the first experiment either the duration of treatment with growth hormone or the amounts given were inadequate to overcome the protection that complete ablation of the thyroid confers on the liver. Only a few benign neoplastic lesions of small size were obtained in this organ. With the larger doses of Experiment II, besides such benign tumours, 3 of the 5 completely thyroidectomized males developed hepatomas the largest of which measured 11 × 10 × 9 mm. This was found in a rat with a body weight of 200 g. and a liver weight of 5·8 g. The animal was killed in the 37th week of the experiment, i.e. at a time when hepatomas develop in intact animals painted with A.F. Signs of liver damage such as increase in connective tissue and proliferation of bile ducts were, on the whole, most pronounced in rats with residual thyroid tissue and least in completely thyroidectomized animals treated with A.F. only. However, the period of survival after the administration of the carcinogen had been stopped varied by several weeks and so did the degree of obesity with concomitant accumulation of fat in the liver. From the material available it is impossible to assess accurately the degree of injury to the liver in the animals of the 3 groups.

---

EXPLANATION OF PLATE

FIG. 1.—Adenoma found in completely thyroidectomized rat injected with growth hormone (Experiment I). Tumour cells are small with slightly basophilic cytoplasm. H. and E. × 150.

FIG. 2.—Hepatoma found in completely thyroidectomized rat injected with growth hormone. (Experiment II). H. and E. × 150.

1

2

Bielschowsky.

As in the previous experiments the majority of the extrahepatic tumours were carcinomas of the meatus acousticus.

### DISCUSSION

The work of Griffin, Richardson, Robertson, O'Neal and Spain (1955), Firminger and Morris (1955), Skoryna (1955) and Hoffman (1956) (quoted from Weisburger and Weisburger, 1957) and of O'Neal and Griffin (1957) has firmly established that carcinogenic azo-dyes as well as 2-aminofluorene derivatives fail to induce liver tumours in hypophysectomized rats. Ablation of the pituitary, however, does not protect other susceptible organs against the tumour-inducing action of N-2-fluorene diacetamide, just as thyroidectomy protects only the liver of rats treated with A.F. Attempts to restore the susceptibility of the liver of hypophysectomized rats to azo-dyes with hormonal preparations of pituitary origin were successful. Griffin *et al.* (1955) found ACTH as well as somatotrophin effective, but the rate of tumour development was slower in hypophysectomized rats injected with either of these hormones than in intact animals. Hoffman (1956) using N-2-fluorenyl diacetamide obtained precancerous lesions in the livers of hypophysectomized rats treated with growth hormone, but not when ACTH was injected.

To judge from the histology of the adenohypophysis the athyreotic rat lacks growth hormone. The results presented in this communication show : it is the lack of this pituitary agent and not the lack of thyroxine that causes the strikingly reduced susceptibility of the liver to A.F. in thyroidectomized rats. This interpretation is supported by the findings of Griffin *et al.* (1955) who could not obtain liver tumours in hypophysectomized rats injected with thyrotrophic hormone.

### SUMMARY

Injection of growth hormone restores the susceptibility of the liver of athyreotic rats to A.F.

My thanks are due to Dr. B. H. Loft of the Frederiksberg Chemical Laboratories, Copenhagen for a generous gift of growth hormone.

### REFERENCES

BIELSCHOWSKY, F. AND HALL, W. H.—(1953) *Brit. J. Cancer*, **7**, 358.
FIRMINGER, H. J. AND MORRIS, H. P.—(1955) *Fed. Proc.*, **14**, 402.
GRIFFIN, A. C., RICHARDSON, L. H., ROBERTSON, C. H., O'NEAL, A. M. AND SPAIN, J. D.—(1955) *J. nat. Cancer Inst.*, **15**, 1623.
HOFFMAN, H. E.—(1956) Dissertation Abstr., 16, 1332. Univ. Microfilm Publ. No. 16847, 122 pp.
O'NEAL, M. A. AND GRIFFIN, A. C.—(1957) *Proc. Amer. Ass. Cancer Res.*, **2**, 236.
SKORYNA, S. C.—(1955) *Proc. Canad. Cancer Res. Conf.*, **1**, 107.
WEISBURGER, E. K. AND WEISBURGER, J. H.—(1957) *Advanc. Cancer Res.*, Vol. V.